# Participatory model building for suicide prevention in Canada

**DOI:** 10.1186/s13033-020-00359-6

**Published:** 2020-04-03

**Authors:** Laura H. Thompson, Justin J. Lang, Brieanne Olibris, Amélie Gauthier-Beaupré, Heather Cook, Dakota Gillies, Heather Orpana

**Affiliations:** 1 Centre for Surveillance and Applied Research, Public Health Agency of Canada, Ottawa, Canada; 2 Department of Community Health Sciences, University of Manitoba, Winnipeg, Canada; 3 Interdisciplinary School of Health Sciences, University of Ottawa, Ottawa, Canada; 4 Centre for Chronic Disease Prevention and Health Equity, Public Health Agency of Canada, Ottawa, Canada; 5 Centre for Health Promotion, Public Health Agency of Canada, Ottawa, Canada; 6 Faculty of Humanities and Social Sciences, Memorial University of Newfoundland, St. John’s, Canada; 7 School of Epidemiology and Public Health, University of Ottawa, Ottawa, Canada

**Keywords:** Suicide, Suicide prevention, Participatory model building workshop, Conceptual model

## Abstract

**Background:**

Suicide is a behaviour that results from a complex interplay of factors, including biological, psychological, social, cultural, and environmental factors, among others. A participatory model building workshop was conducted with fifteen employees working in suicide prevention at a federal public health organization to develop a conceptual model illustrating the interconnections between such factors. Through this process, knowledge emerged from participants and consensus building occurred, leading to the development of a conceptual model that is useful to organize and communicate the complex interrelationships between factors related to suicide.

**Methods:**

A model building script was developed for the facilitators to lead the participants through a series of group and individual activities that were designed to elicit participants’ implicit models of risk and protective factors for suicide in Canada. Participants were divided into three groups and tasked with drawing the relationships between factors associated with suicide over a simplified suicide process model. Participants were also tasked with listing prevention levers that are in use in Canada and/or described in the scientific literature.

**Results:**

Through the workshop, risk and prevention factors and prevention levers were listed and a conceptual model was drafted. Several “lessons learned” which could improve future workshops were generated through reflection on the process.

**Conclusions:**

This workshop yielded a helpful conceptual model contextualising upstream factors that can be used to better understand suicide prevention efforts in Canada.

## Introduction

In 2015, 2.5% of the Canadian population 15 years and older reported having thoughts of suicide, 0.8% had made a plan, and 0.4% had attempted suicide in the previous 12 months [1]. Suicide is a behaviour that results from a complex interplay of factors, including biological, psychological, social, cultural, and environmental factors, among others [2, 3]. Multiple contributory factors that may lead to suicide are as diverse and multi-faceted as economic crises [4], access to means [5], and media reporting of suicide [6], and may be influenced by intra-individual factors such as pain and illness [7, 8]; hopelessness and guilt [9]; and problem solving deficits [10]. All of these contributing factors may vary by age, gender and other sociodemographic characteristics [1]. In response to this complex public health issue in Canada, *An Act Respecting a Federal Framework for Suicide Prevention* became law in Canada in December 2012 and was followed by the Federal Framework on Suicide Prevention in 2016 [11]. The objectives of “The Framework” are to reduce stigma and raise public awareness, connect Canadians to information and resources, and accelerate the use of research and innovation in suicide prevention [12].

Given the complexity of suicide, a range of prevention initiatives aimed at reducing risk among the general population (e.g. universal prevention such as installing barriers on bridges), subpopulations at heightened risk (e.g. selective prevention such as training first responders), and among individuals at high risk of suicide (e.g. indicated prevention such as treatment of mental illness) [2, 13] are generally used, although the strength of the evidence for particular prevention strategies may vary [2, 14]. In a number of areas of public health, conceptual models have been used to map out complex issues to bring to light the interconnectedness of prevention efforts. For instance, Allender et al. [15] developed a conceptual model of childhood obesity to create a multi-faceted understanding of how factors as diverse as pet ownership, marketing of processed foods, and income inequality interact dynamically to influence obesity levels. The development of these conceptual models provides an opportunity for knowledge to emerge and for consensus to be built while generating a tool that may be used to organize and communicate the complex factors and their interrelationships that influence an outcome of interest. These models can be used to identify leverage points and also important gaps in prevention, services, or policy.

One approach to developing a conceptual model of a complex public health issue is through a participatory model building workshop. Participatory model building has been used since the late 1960s to investigate complex issues as diverse as water resource management [16], sustainable development [17], and the relationship between primary health care and population health [18]. Rather than relying solely on evidence derived from traditional scientific research, this approach allows for a greater breadth and complexity of knowledge to be incorporated, which is important for the identification of the practical leverage points that are necessary to address complex issues [19] These workshops provide an opportunity for participants to develop shared understandings of a problem of interest, uncovering hidden assumptions, revealing differences in the language that people are using, and ultimately leading to intervention approaches that may be more effective and more readily implemented [20–23].

Participatory model building workshops provide an opportunity for stakeholder engagement and to capture the insights of those who deal with the problem of focus on a day-to-day basis [24], leading to the development of models that may more accurately reflect the real world [25]. A failure to consider real world complexities may result in the implementation or continuation of interventions that fail to achieve the desired outcomes or even worsen problems [26]. An emerging application of participatory model building is for quantitative system dynamics modelling, which may be used to develop more accurate simulations of complex systems [26–28].

The objective of this paper is to describe the process of implementing a participatory model building workshop to develop a new conceptual model for suicide prevention in Canada. The aims of the model building workshop were to (1) generate a conceptual model that expanded beyond conventional models of suicide, by incorporating upstream risk and protective factors that act at the level of individuals, families, communities, and societies, and (2) identify the prevention levers that may operate upstream and across different intersecting societal structures and systems in the Canadian context. The resulting model could help inform future public health efforts around suicide prevention, guide future research, and inform micro-simulations and modelling efforts aimed at quantifying the impact of population-based interventions [29].

## Methods

### Workshop participants and context

Individuals invited to participate in the workshop were federal public servants who worked at the Public Health Agency of Canada (PHAC). PHAC is a federal agency with the mission to “protect the health of Canadians through leadership, partnership, innovation and action in public health.” PHAC is responsible for policy and program activities relating to suicide prevention, surveillance and applied research related to suicide and its prevention, and coordinates the Federal Framework on Suicide Prevention across the federal government. As such, hosting a participatory model building workshop at PHAC provides access to individuals with diverse real-world experiences and knowledge in the day-to-day national activities aimed at suicide prevention.

More specifically, the participatory model building workshop was conducted with PHAC employees on June 19, 2018. Two months prior to the workshop a facilitator sent invitations to twenty employees who were selected because of their roles in policy and program activities related to suicide prevention. Potential participants were encouraged to invite other employees who had experience in suicide prevention in an effort to include all employees with relevant experience and knowledge. Those who declined the invitation generally did so because another person from their unit had also been invited and planned to attend the workshop. Participants were asked not to do any preparatory work prior to the workshop, and were told that their working knowledge in suicide prevention would be enough to contribute meaningfully to the workshop. Specific participant details are withheld to maintain participant anonymity.

### Pre workshop preparation

**Fig. 1:**
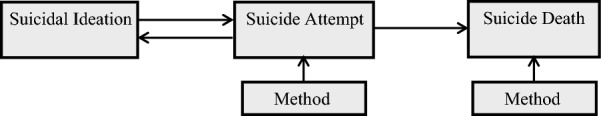
Simplified suicide process model

In the 3 months leading up to the workshop, a facilitator’s script was developed to walk participants through the activities laid out in the agenda. Activities were organized in a way to elicit rich participant responses and engagement. This script was developed by a core team of four individuals and was informed by published peer-reviewed articles describing other participatory model building processes [20, 21, 30]. A presentation was developed in alignment with the script to provide relevant visual prompts for participants. Participants were provided with a printed package containing the agenda; a simplified suicide process model (Fig. 1) [29]; a pre-existing list of risk and protective factors for suicide prevention [1] developed in 2015; a diagram from the World Health Organization showing how key risk factors for suicide align with relevant interventions (as an example of how to match interventions to risk and protective factors during the workshop) [31]; and the evaluation form. The planning team prepared four 50″ × 30″ pieces of paper and print outs of the simplified suicide process model in 32 point font to facilitate groups placing risk and protective factors onto the simplified suicide process model [29]. An additional 50″x30″ piece of paper with the process model was prepared for the subsequent discussion to allow for further diagramming, if necessary. The planning team piloted the group work activity 2 weeks prior to the workshop in order to test and refine the approach and prepared materials. The script was also read aloud to the planning team for further feedback.

### During the during the workshop

Two individuals facilitated over the course of the workshop day, and two other team members took notes and circulated during group work to answer questions. Another team member took photographs with the groups’ permission. Coffee and baked goods were available as participants walked into the room, and as part of the welcoming message facilitators emphasized that participants were welcome to offer their thoughts, comments, and ideas at any time during the day—or leave if they needed to. Following participant introductions, the purpose of the workshop was defined and an example conceptual model was shown. To set the stage for the inclusion of a sex and gender-plus lens, essential in light of the fact that some groups of men, women, boys, girls, and gender-diverse individuals are differently affected by suicide [11], participants watched a short video on the topic [32]. Afterwards, the morning’s work began with a review of a simplified suicide process model (Fig. 1), around which the draft of an enhanced conceptual model would be built over the course of the day.

Next, the risk and protective factors included in the *Suicide Indicator Framework* [1], which was developed through a collaborative consultative process in 2015, were reviewed and participants suggested further factors to add. Following this exercise, participants were assigned to three groups and each group was asked to place the risk and protective factors that they considered to be the most relevant onto the simplified suicide process model. The similarities and differences of the draft conceptual models developed by the three groups were then discussed with the entire group and a facilitator talked through the key concepts that had emerged and recorded them onto a draft consolidated conceptual model. The workshop resumed after lunch with a discussion of prevention levers. Participants were encouraged to consider levers that operate at all levels, including interventions that target individuals, families, and communities or all of society. Prevention levers could also take different forms, including services to individuals, education, guidelines, and regulations, and they could impact risk or protective factors or the suicide process itself. Participants were also encouraged to include prevention levers that were relevant in Canada but have not been implemented, as their inclusion at the workshop could shape future work. The intention was to overlay prevention levers onto the draft conceptual model that was developed over the course of the morning and ask participants to identify their top three priority areas, but time and participant energy levels did not allow for this.

### Post workshop debrief

Following the workshop, the planning team reviewed the list of risk and protective factors and the list of prevention levers which were developed over the course of the workshop and worked to streamline these lists by grouping similar concepts. Next, this revised and consolidated list of risk and protective factors was overlaid onto the simplified suicide process model in accordance with the structure of the three draft models developed during group work, generating a “revised draft model”. The revised draft model was circulated to all workshop participants for their feedback and approval.

A printed participant evaluation form was provided to all participants at the workshop, and also circulated to all participants by e-mail 1 day later. A researcher identified key quotes provided by the participants through the evaluation forms to help identify lessons learned.

One of the key planning team members interviewed other planning team members to elicit their feedback about the methods and “lessons learned”. These interviews occurred between July 9, 2018 and July 17, 2018. Interviewees were assured that their names would not be attached to any specific comments but rather all feedback would be compiled into one document. Open ended questions such as “what worked and what did not work?”, “what did we do well?” and “what could we do better next time?” were used to direct the conversations. A rapid thematic analysis was conducted by one researcher to identify common themes that arose across all the interviewees. Common themes were used to help identify lessons learned.

Research ethics board approval was not required for this model building workshop because workshop participants were not acting in the role of research subjects. The Standards for Reporting Qualitative Research checklist was completed for this article.

## Results

### Workshop participation

The workshop began with fifteen participants from six units within PHAC: 4 from suicide surveillance, 6 from suicide prevention, 1 from family violence prevention, 1 from seniors’ health, 1 from mental health promotion, and 2 from sex and gender-based analysis. As the workshop carried on throughout the day some participants had to leave due to illness or urgent work matters. By the end of the day a core group of approximately ten participants had been engaged throughout the entire workshop.

### Development of preliminary draft models

Participants generated a long list of risk and protective factors which operate at individual-, family-, community-, and societal-levels (available in Additional file 1: Table S1), building from the list included in the 2015 *Suicide Indicator Framework* [1]. Additions that participants made to the list included factors related to discrimination and oppression, such as racism, homophobia, and systemic racism. Individual factors were summarized into aggregate categories such ‘stressful events and circumstances’ and ‘family relationships’ (Table 1).

**Table 1:** Reduced list of risk and protective factors

Individual-level risk and protective factors
Individual (personal) attributes
Relationships and connectedness
Stressful events and circumstances
Resilience and coping
Health and illness
Substance use
Screen time and media
Interactions with legal system
Access to suicide means
Family-level risk and protective factors
Family relationships
Family history of illness, substance use, suicide, trauma
Family violence
Community-level risk and protective factors
Social support and community
Environment
Violence
Community-level trauma and suicide
Mental health services
Societal risk and protective factors
Stigma, discrimination and oppression
Mental health infrastructure
Structural factors (general)

**Fig. 2:**
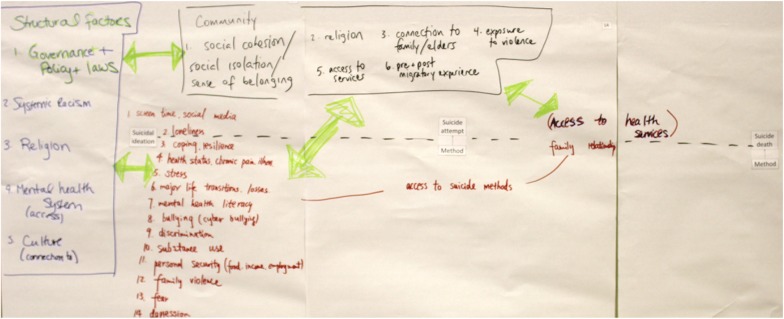
Group work output example

Next, participants formed three groups and each group wrote the risk and protective factors that they considered most important onto the simplified suicide process model (Fig. 2). Participants identified several challenges in doing this: (1) determining where to place factors on the suicide process model, (2) how to identify on the model the level at which a factor operates (i.e. individual, family, community, societal), and (3) how many of the risk and protective factors to place on the model and whether to group them. Figure 2 shows how one of the three groups chose to handle these challenges, grouping and colour-coding risk and protective factors using three categories of levels: individual-level, community-level, and structural-level.

**Fig. 3:**
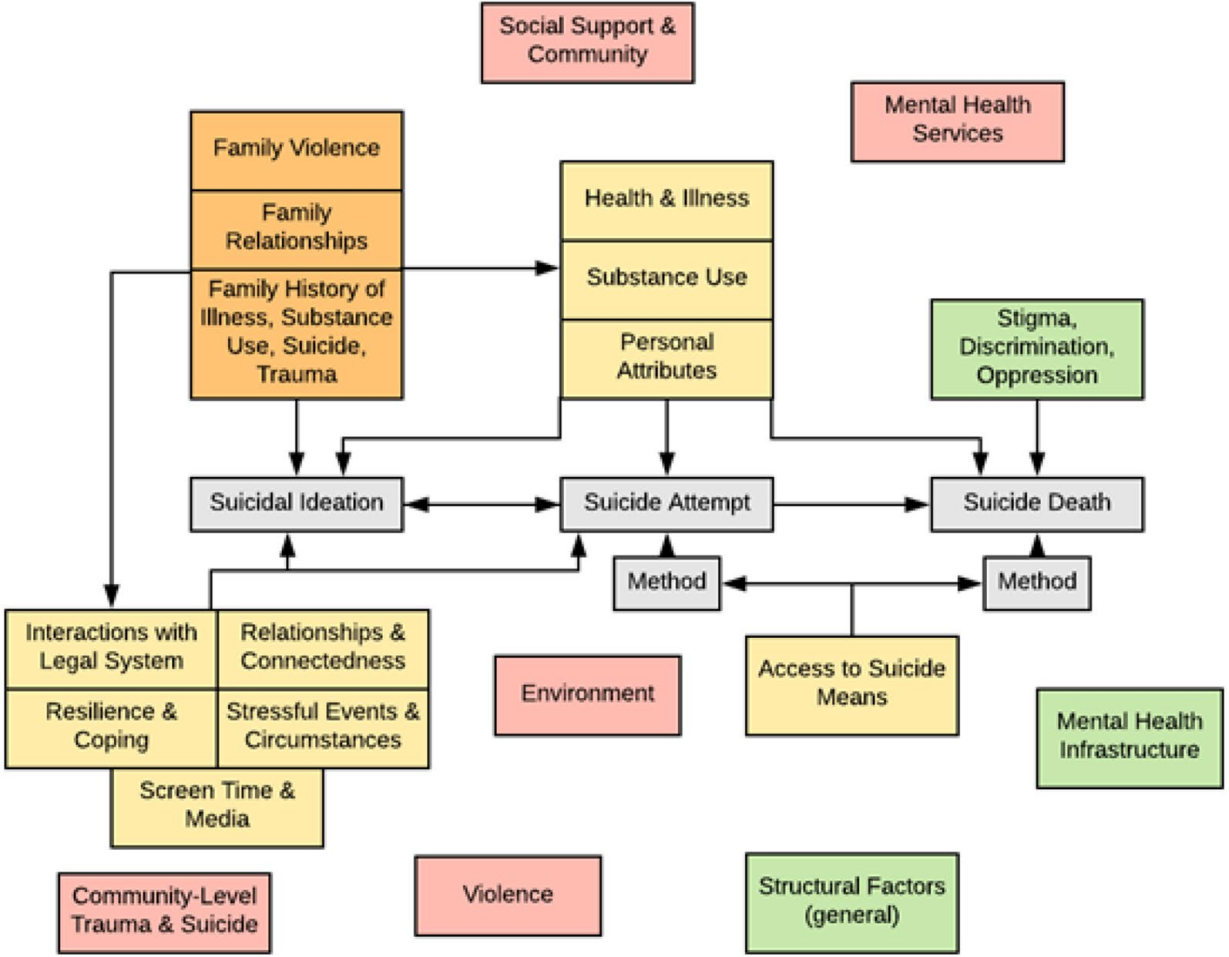
Consolidated enhanced draft conceptual model of risk and protective factors

The three draft models were combined into a consolidated draft conceptual model which was refined by the planning team following the workshop, and validated by workshop participants by sharing the model for comment through e-mail. This resulted in a final consolidated draft conceptual model (Fig. 3) which shows the group’s conceptualisation of how risk and protective factors at all levels influence each other and the suicide process. Although community and societal factors are not explicitly linked using arrows in the draft model as a simplification, their dynamic and continuous influence on all aspects of the draft model is implied. Additionally, within each factor, sex, gender and diversity considerations are essential.

The complete list of prevention levers that were mentioned during the workshop are presented in the original language used and captured by the note taker during the workshop in Additional file: Table S2. For the purpose of simplification, these levers have been organized according to the stage of the suicide process where they operate (primary prevention, intervention, and support for survivors [postvention]) and individual levers have been summarized logically into larger categories such as ‘health, social, and community programs’ and ‘guidelines and evidence’, which may operate at all three points of the suicide process (Table 2). As prevention levers are not always bounded clearly into just one category, care was taken to apply the best-fitting category.

**Table 2:** Reduced list of prevention levers

Early/primary prevention (before suicide risk)
Mental health promotion
Health, social and community programs
Structural change and social justice
Guidelines and evidence
Intervention
Health, social and community programs
Health care structure
Guidelines and evidence
Support for survivors (postvention)
Mental health services
Guidelines and evidence

### Evaluation and lessons learned

Only four participant evaluation forms were received. These responses were nearly all positive, with a few helpful comments:

*“*It was a great 1 day of discussion on suicide prevention and what can be done. There was a lot to think and a lot to cover in 1 day, if time allocated cannot be changed, perhaps give some reading ahead of time so people come prepared about what to expect and what to provide input on. For example, some more time to discuss factors in the morning would have been more useful. As well, the voice of children and youth was missing and it might be useful to encourage their participation—especially in levers for prevention of suicide.” (Participant 2)

*“*Splitting this workshop into a 2-day session would be extremely beneficial as it would allow participants to reflect and come back with fresh eyes on the 2nd day. The day was very packed and made it difficult for us to go through the afternoon activity finding a way to lighten the session or, as mentioned above, splitting into a 2-day workshop could be helpful.” (Participant 4)

One member of the planning team observed the workshop proceedings and noted that not all of the participants were fully engaged throughout the day. The waning energy levels, particularly after the lengthy discussion about prevention levers, were also observed.

There were several lessons learned in implementing our participatory model building workshop. The planning team reported that it was important to have food and drinks available all day during the workshop and to have a long break for lunch. During introductions, participants were asked to share “what they had for breakfast” to break the ice and this was seen as a non-intrusive and fun approach. The contributions of our relatively large planning team were seen as very helpful, and allowed for our agility on the day of the workshop. Piloting the script and group work activities and ensuring that the team was quite familiar with them was seen as a key to success, as was the team’s flexibility to adapt to the needs of the group over the course of the workshop.

Team members suggested in future dividing a workshop over two half days to ensure higher energy levels for the entire program. The use of a meeting room in a building other than the one that participants work in could help to reduce distractions over the course of the workshop. Also, there may have been a contradiction in asking participants to do no preparatory work and therefore bring only their day to day working knowledge while still asking them to think beyond their mandate and at other levels. However, the goal of the workshop was to provide an opportunity for the participants’ own knowledge to emerge. Facilitation requires skill to ensure that participants have the necessary information to perform the requested activity while not providing too much information or taking the workshop off track. Additionally, care should be taken to restrict what is provided to participants to only what is absolutely necessary. For instance, providing an example conceptual model led participants to discuss that specific example model, which took time and energy away from the workshop content and may have influenced workshop outcomes. Listing risk and protective factors and prevention factors took more time and discussion than was expected. The time and energy that it takes to perform each individual activity of a workshop needs to be carefully considered in advance and aligned carefully with workshop objectives.

## Discussion

Since the 1990s there have increasingly been calls for epidemiologists and public health practitioners to consider explanatory factors that extend beyond the behaviours and biology of individuals, with the recognition that a wide range of complex factors interact dynamically in a system to produce patterns of ill health in populations [33, 34]. More recently, developing a greater understanding of the macro-level determinants of health and creating methodological innovation capable of “tackling the central questions about how social circumstances shape the health of populations” have been described as two formative next steps in the advancement of epidemiology ([34], p. 845). While data are most readily available for individual-level factors that are more easily quantifiable and most proximate to suicide, such as thoughts of suicide (obtained through cross-sectional surveys) and self-harm related hospitalizations (obtained through hospital administrative databases), the consideration of such factors alone does not adequately capture the complexity of suicide and suicide-related behaviour [35]. The participatory model building workshop was used to capture the understandings of employees who work in suicide prevention or related areas in a federal public health organization, eliciting useful information about upstream risk and protective factors [19]. The addition of factors related to discrimination and oppression to the pre-existing list of risk and protective factors is important, particularly given the higher rates of suicide among some Indigenous populations in Canada [36, 37]. Together, these factors interrelate and operate at multiple levels, and the resulting draft conceptual model is an initial attempt to articulate this. It is recognized that this “enhanced” draft conceptual model is not a true representation of reality, but reflects the participants’ mental models of suicide and suicide prevention. The draft conceptual model can be used as a starting point for discussion, empirical testing, refinement, and adaptation for specific subpopulation groups.

While many of the participants enjoyed an opportunity to share their knowledge, it was challenging to determine the best way to structure a model that is composed of so many diverse factors which interact with each other in a multitude of ways. However, the challenging nature of this was expected, as “visualizing complexity is part of the aim of the exercise” [15]. Ultimately, the workshop planning team worked to consolidate and simplify the drafts of the model developed by workshop participants in order to capture the overarching themes while generating a useful tool to communicate concepts and identify upstream factors (macro-level determinants of health) of importance.

The draft conceptual model developed in this study is the first of its kind that represents a unique Canadian perspective on suicide behaviours and prevention that goes beyond what is currently available in the scientific literature. For instance, many conceptual models of suicide focus on particular subpopulations, such as those who live in rural settings [38] or are members of the military [39], typically emphasizing interactions between predisposing and precipitating factors [3] such as connectedness [40] or physical illness [41]. Our draft model, instead, is a reflection of the understandings of the participants in our model-building workshop, intended to apply broadly to the general population of Canada and incorporates risk and protective factors, based on the social determinants of health, at multiple levels. At the level of the individual, intersecting factors such as gender, religion, disability, and culture which may increase risk or provide protection through a variety of pathways are included [42–45]. At the level of the family, research has indicated, for example, that family connectedness is a strong predictor of positive mental health outcomes among transgender youth [46], while child maltreatment is associated with adolescent suicidal behaviours [47]. Factors such as suicide contagion [48], school environments [49], and work environments (e.g. among first responders [50]) play out as risk and protective factors at the community level. At the societal level, our draft model incorporates means restriction laws [51], systemic racism [52, 53], and media reporting guidelines [6], among others, as important and intersecting risk and protective factors for suicide. The US Centers for Disease Control and Prevention has also developed a comprehensive model which they termed *Socio*-*Ecological Suicide Prevention Model* (SESPM), with accompanying recommendations for the establishment of multi-level social-ecologically based suicide prevention programs [38]. The model presented here is based on the understanding of those who participated in the workshop; as such, a workshop with different participants may have yielded a different model. Discussion and refinement of these models is invited [38].

### Implications and future research

There are several implications of the conceptual model developed through our participatory model building workshop. First, the model provides the unique perspective of the participant’s perceptions of risk and protective factors that influence suicide behaviours in the Canadian context. As such, this model can help guide decision makers as they develop suicide prevention activities by identifying a broader range of risk and protective factors that may be important at different stages of the suicide process. Second, this model can inform suicide prevention research in areas where there is not currently a large body of evidence. Third, we provide a list of prevention levers identified through the workshop. While not exhaustive, this list could serve as a starting point for further discussion of entry point to suicide prevention. Last, our model is considered a draft conceptual model that could still be further refined. We hope that our model, developed through a participatory model building approach, stimulates further consideration of a systems approach to suicide prevention.

Not all of the planned activities could occur over the course of the day, and as a result the prevention levers could not be included on the final “enhanced” draft conceptual model. Future research could further develop our draft conceptual model though a causal loop diagram that captures complexity and the reciprocal relationships between concepts. It may also be possible to use this draft conceptual model as a starting point for other modelling approaches, such as system dynamics or agent based modelling. It would also be important to further refine our draft conceptual model—which reflects the perspectives of the workshop participants—by consulting a broader group of individuals and organizations.

## Limitations

The results of this study are subject to several limitations. The results of this model building workshop are limited to the perspectives of staff of a federal public health organization that are policy analysts, program staff and surveillance analysts and did not include suicide prevention service providers or other individuals who work at regional or local levels and who may have different knowledge and perspectives. Members of the community were also not included as participants in this workshop. While people with lived experienced were not explicitly included among the participants, it is possible that some workshop participants also brought this perspective to the workshop. The draft model that was developed over the course of the day was limited by time and the energy of the workshop participants. This draft model was developed specifically with regards to the general population and further targeted work and consultation would be required to generate models appropriate for specific populations, particularly those which experience inequalities in suicide risk due to factors including structural violence, marginalization, and historical trauma.

## Conclusions

The participatory model building workshop provided an opportunity for staff to share their knowledge of suicide and suicide prevention in Canada through a structured approach. This workshop yielded a draft conceptual model that contextualised upstream factors that can be used to better understand suicide prevention efforts in Canada. The conceptual framework that emerged from this workshop could be used to inform suicide prevention research in the Canadian context. It also presents an excellent opportunity to bridge the gap between policy, programs and research.

## Supplementary information

**Additional file 1: Table S1.** Original list of risk and protective factor categories. **Table S2.** Original list of prevention lever categories.

## Data Availability

The information collected during the current study is available from the corresponding author on reasonable request.

## Abbreviation

SESPM: Socio-ecological suicide prevention model
