# Supplementary material for: Participatory model building for suicide prevention in Canada
Source: Int J Ment Health Syst. 2020 Apr 3;14:27. doi: 10.1186/s13033-020-00359-6 (PMC7118927; doi:10.1186/s13033-020-00359-6)
Supplement: Supplementary file 1 — Additional file 1: Table S1. Original list of risk and protective factor categories. Table S2. Original list of prevention lever categories. [file 13033_2020_359_MOESM1_ESM.docx]

**Supplementary Table 1. Original List of Risk and Protective Factor Categories**

| **Individual-Level Risk and Protective Factors** | |
| --- | --- |
| Individual (Personal) Attributes | SES, age, sex, gender, education |
|  | Personality |
|  | Spirituality, religion, culture |
| Relationships & Connectedness | Relationship quality, loneliness, isolation |
|  | Connections to family, role models, elders |
|  | Connection to culture, sense of belonging, community engagement |
| Stressful Events & Circumstances | Major life transitions, losses, grief, trauma, (pre)migratory trauma |
|  | Security (e.g. food, personal security) |
|  | Exposure to violence, suicide |
|  | Loss, grief, loss of meaning/hope |
|  | Experience of stress |
| Resilience & Coping | Positive mental health, resilience, coping |
|  | Help seeking behaviour, mental health literacy |
| Health & Illness | Health status, chronic pain, illness, sensory impairment |
|  | Sleep disturbances |
|  | Mental illness |
| Substance Use | Problematic substance use |
| Screen time and media | Screen time, social media use |
| Interactions with legal system | Interactions with legal system |
| Access to suicide means | Individual access to suicide means |
| Family-Level Risk and Protective Factors | |
| Family Relationships | Family relationships, attachment/parenting style |
| Family History of Illness, Substance Use, Suicide, Trauma | Family history of mental illness, problematic substance use, suicide-related behaviour, trauma |
| Family Violence | Family violence |
|  | Child protection involvement |
| Community-Level Risk and Protective Factors | |
| Social Support & Community | Social support and social cohesion |
| Environment | School or work environment |
| Violence | Violence, bullying, cyber bullying |
| Community-Level Trauma & Suicide | Trauma, loss |
|  | Suicide contagion/clusters |
| Mental Health Services | Accessible mental health services (e.g. language, knowledge/skills of practitioners) |
| Societal Risk and Protective Factors | |
| Stigma, Discrimination & Oppression | Stigma generally and related to suicide |
|  | Racism, homophobia, biphobia, transphobia |
|  | Systemic racism |
|  | Discrimination, oppression |
| Mental Health Infrastructure | Mental health service access |
|  | Mental health literacy |
| Structural Factors (general) | Structural factors (e.g. policy, laws, governance) generally and related to suicide |
|  | Suicide media reporting guidelines (and adherence) |
|  | Laws related to means and means restriction (e.g. firearm regulations, poison control) |

**Supplementary Table 2. Original List of Prevention Lever Categories**

| Early/Primary Prevention (Before Suicide Risk) | |
| --- | --- |
| Mental Health Promotion | Positive mental health promotion |
|  | Mental health literacy promotion for children and youth; older adults; caregivers/families |
|  | Supportive environments for children and youth; older adults; caregivers/families with pathways to help (e.g. guidance counsellors) |
| Health, Social & Community Programs | Community champions |
|  | Actions to reduce the problematic use of substances |
|  | Social policies and programs that address poverty, employment/EI, safety nets |
|  | Actions that decrease stigma |
|  | Prevention of violence and abuse |
|  | Address upstream determinants of health – interventions for vulnerable groups – social cohesion e.g. Elders and different parts of the community brought together to develop response |
| Structural Change & Justice | Community design, policy, events, built environment, car-centricity, security, safety |
|  | State-inflicted violence – policy levers – to identify policies/structures/behaviours that systematically disadvantage specific groups |
|  | Restorative or alternative justice practices |
| Guidelines & Evidence | Guidelines – health practitioners, media, etc |
|  | Generate and provide evidence to policy makers; health in all policies approach (WHO) where health sector is not a leader but a steward: labour, economic policies, etc, bring in mental health protective factors |
| Intervention | |
| Health, Social & Community Programs | Gate-keeper training – so practitioners, etc can better understand what people really go through |
|  | Help-line, crisis-line, proactive check-in services e.g. Telecheck to check in |
|  | Facilitate long-term disability options for depression |
| Health Care Structure | Comprehensive, broad approach and complex issues, eg for trauma recovery it would be multi-dimensional |
|  | Health promotion in context of primary care; bridge atomized approaches to mental health promotion services; bridge fed/PT roles |
|  | Decentralization of care – referrals; follow-up following interaction with the healthcare system – augment referral system |
|  | Health care practitioner supply to match the population – culture, ethnicity, language |
| Guidelines & Evidence | Who are priority populations when we talk about suicide; how is this information recorded; where are the needs |
| Support for Survivors (Postvention) | |
| Mental Health Services | Community-driven follow-up care |
|  | Population-specific guides which can reach those who need e.g. outreach to funeral homes? Who is in the best position to reach? Best approach to distribution |
| Guidelines & Evidence | Medical assistance in dying – clarification of issue – later life perspectives – when to intervene, preconceptions, training practitioners |
|  | Canadian standards for messaging and reporting, cross-sectoral |
